# Renal Health Benefits of Rural City Planning in Japan

**DOI:** 10.3389/fneph.2022.916308

**Published:** 2022-06-15

**Authors:** Kei Nagai, Daniel Koo Yuk Cheong, Atsushi Ueda

**Affiliations:** ^1^ Department of Nephrology, Hitachi General Hospital, Hitachi, Japan; ^2^ Department of Nephrology, Faculty of Medicine, University of Tsukuba, Tsukuba, Japan; ^3^ Center for Inflammatory Diseases, Department of Medicine, Monash University, Clayton, VIC, Australia

**Keywords:** chronic kidney disease, active transport, exercise, pedometer, public transport, city planning

## Abstract

Progression of chronic kidney disease (CKD) is a substantial threat because it is associated with reduced healthy life expectancy and quality of life, and increase in economic burden. Research indicates people with nondialysis CKD often have lower physical functioning and that improvement of physical activity may contribute to maintaining renal health. Another issue with the current treatment of CKD is that the synergistic effects of rural depopulation due to aging and uncontrolled rural city sprawling will increase the number of under-served healthcare areas. To ensure the quality of renal health care, hospital integration is desirable, under the condition of reconstruction of the public transport system for physically and socially vulnerable people. Recently, medical and non-medical scientists advocate the challenge of city planning for population health. The links between city design and health such as cardiovascular disease, obesity, type 2 diabetes and mental disorders, have been widely studied, except for renal health. Based on our experience in a Kidney and Lifestyle-related Disease Center, we propose the idea that city planning be prioritized to improve renal health through two main streams: 1) Improve physical status by use of public and active transportation including daily walking and cycling; and 2) Equal accessibility to renal health services. Many countries, including Japan, have enacted plans and public policy initiatives that encourage increased levels of physical activity. We should focus on the impact of such movement on renal as well as general health.

## Introduction

The number of individuals with chronic kidney disease (CKD) has steadily increased worldwide (1–3). Japan is no exception, as the prevalence of CKD has also increased with the aging of the population (3). Progression of CKD is a substantial threat, because it is associated with reduced healthy life expectancy and quality of life, and increase in economic burden (4, 5). In addition, people with nondialysis CKD often have lower physical functioning compared to that of the general population (6). Lack of physical capabilities may lead to physical inactivity, which is also a growing threat to public health (7). Physical inactivity significantly increases the risk of mortality and a number of non-communicable diseases including obesity and diabetes (8). Therefore, physical inactivity is considered one of the major preventable risk factors for mortality in Japanese adults (9). Recently, in an international collaborative study of CKD patients without dialysis therapy, there was significantly higher mortality (hazard ratio [HR] = 1.62) and a trend toward more rapid CKD progression (HR = 1.17) among those not physically active compared to those who were highly active (10). These results imply improvement of physical activity could contribute to maintaining renal health. Daily activity level is usually correlated positively with the number of steps taken and negatively correlated to sitting time. As Japan’s sprawling cities expand from their centers, the donut phenomenon increases and privately owned motorized vehicles have become the main mode of transport over public options. As public transport use has declined, the opportunities for walking on a daily basis have also declined, leading to lower step counts.

**Figure 1:**
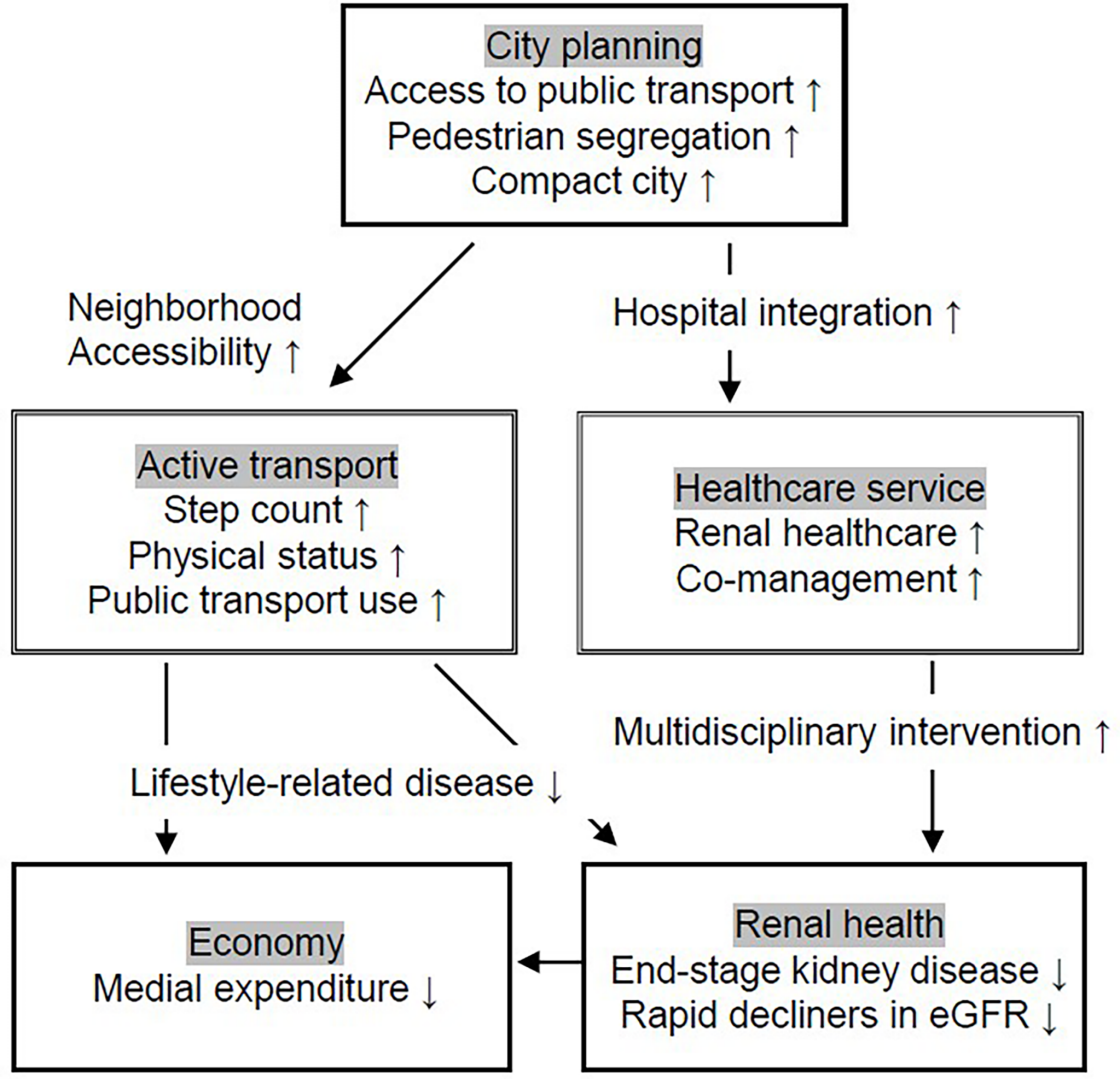
Need of city planning to improve renal health through promotion of active transportation and hospital integration. We propose the idea that city planning is prioritized to improve renal health and subsequently reduce medical expenditure. The two main streams are: 1) Improve physical status by use of public and active transportation including walking and cycling; and 2) Accessibility to renal health service.

Concurrently, the synergistic effects of rural depopulation due to aging and uncontrolled rural city sprawling will increase the number of under-served healthcare areas. In these areas, it is difficult to receive specialized treatment on a regular basis, much less access it by public transportation and active transport. Regarding renal healthcare, since the number of nephrologists is limited, integrating hospitals is necessary. This will ensure the likelihood of both the management of CKD patients, and the cooperation between core hospitals and general physicians, having a favorable impact on renal function (11–13). However, there is a real danger of promoting a disparity in the quality and quantity in medical care if hospitals are integrated without cooperation between hospitals and general physicians, and without reconstruction of the public transport system for physically and socially vulnerable people. In order to minimize these risks and to improve renal health outcomes, we here propose the idea to reform the city planning policy upstream of the healthcare field (**Figure 1**).

## The Need of City Planning to Improve Renal Health: Transportation and Integration of Healthcare

Before the mid-20th century, public transportation was the dominant mode of transport. Naturally, the economic and cultural center of the city surrounded this system. However, since then, widespread use of private vehicles arose. In the 21st century, private vehicle use in regional areas has seen train stations and the surrounding buildings shuttered, forcing the necessary infrastructure and stores to be concentrated along main traffic roads. Whilst large, completed, densely populated cities often experience little impact, rapid urbanization in regional areas has unfortunately resulted in many buildings, which were once bustling in the past around regional city stations being abandoned.

Meanwhile, private vehicles have become daily necessities in such regional cities which further advance changes in the city structure. Japan’s local cities are seeing fewer people use stores and facilities around stations. The lower usage caused the public transport system to decrease their frequency of transits, which in turn, made the system more inconvenient and even less used. The two main downstream effects brought on by this type of change in regional city structure are reduction in human physical activity and poor access to adequate medical care (**Figure 1**).

Recently, medical and non-medical scientists have advocated a challenge to city planning for improvement on their population’s health. The main idea considered to achieve this goal was to focus on directly affecting the need for private motorized vehicles. By determining the location of housing in relation to employment, education, and the services required for daily life, including healthcare, we could discourage the use of private vehicles and implement designs to promote walking, bicycling and use of public transportation (14). In this context, compact cities—cities of short distances enabled by higher densities—can offer advantages and slight disadvantages in relation to health and well-being. They provide greater access to destinations, enable the use of efficient public transport systems, and provide walkable access to a wider range of facilities. Moreover, the links between compact city features and health such as cardiovascular disease, obesity, type 2 diabetes and mental disorders have been widely studied, except for renal health (15).

**Figure 2:**
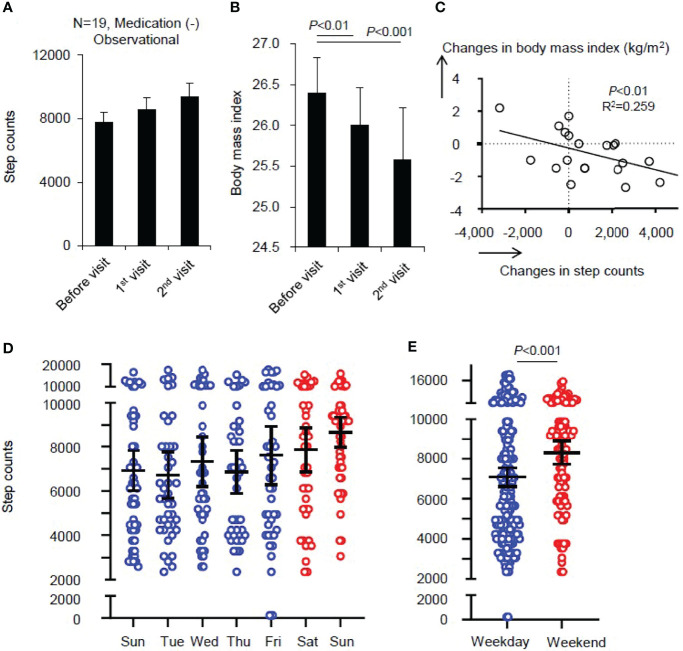
Changes in step counts and body mass index by multidisciplinary education without medication for obese population. In the Kidney and Lifestyle-related Disease Center in Hitachi, Japan, we developed a multidisciplinary care scheme to overcome lifestyle-related disease, comprised of dieticians, physical therapists, nurses and doctors. The participants were mildly obese with hyperlipidemia, diabetes or hypertension. We introduced a short-term guidance program for improving lifestyle without medication. We observed changes in the number of average daily step count through a pedometer, and physical status, among 19 volunteers with mild obesity. Step counts increased and body mass index was significantly reduced, and they were correlated **(A–C)**. We found recreational walking is the dominant contributor for increasing step counts **(D, E)**. Median and standard error are presented as bars and error. Median values were compared by the Mann-Whitney test and the correlation was examined by the Spearman test.

In our Kidney and Lifestyle-related Disease Center, we aim to develop a regional medical system to overcome lifestyle-related disease through multidisciplinary care by dieticians, physical therapists, nurses and doctors (16). To demonstrate this, we provided mildly obese patients with hyperlipidemia, diabetes or hypertension a short-term guidance program. The program consisted of outpatient visits at one and two months after the initial visit without medication. We observed changes in the number of average daily step count, through a pedometer (Medi-walk, Terumo Co., Tokyo, Japan), and physical status, among 19 volunteers with mild obesity who visited our department. Median values of step counts and body mass index were compared by the Mann-Whitney test and the correlation between step counts and body mass index was examined by the Spearman test. Step-counts increased from 7,747 to 9,384 (*P*=0.11) and their median body mass index decreased from 26.4 to 25.6 kg/m^2^ (*P*<0.001). (**Figures 2A, B**). These changes in step counts and body mass index were significantly correlated (*r* ^2 =^ 0.259, *P*<0.01) (**Figure 2C**), suggesting that educational programs without any medication may have a positive effect in the improvement of obesity through increased physical activity. We further observed the mean daily steps on weekends (Saturday and Sunday) were more than those on weekdays (Monday to Friday), based on a total of 52 weeks of step count data for the participants (**Figures 2D, E**). These results suggest recreational walking is a dominant contributor for increasing step counts, though they were from an uncontrolled and sparsely manipulated observation, so the level of evidence is still weak. Regarding evidence in step count and health issue has been definitive because a series of prospective cohort studies have shown that an increase in the number of daily walks reduces the incidence of obesity, diabetes (17–19), and cardiovascular disease (20–22). Well-designed cities are also known to increase walk counts (14, 23). On the other hand, several pieces of evidence demonstrate that neighborhood desirability such as levels of traffic and safety from crime and access to public open space is inconsistently associated with recreational walking (24). Similarly, the evidence that recreational exercise effects health also is inconsistently described. For physical activity to lead to better health, we consider it would be important to ensure that people get enough exercise during the week through commuting to work, school, and other daily activities that make up the majority of weekdays. Currently, commuting in rural areas is mainly dependent on private motorized vehicles. Our next challenge is to build upon the increase in step counts gained through education and raise it further by incorporating active transport and public transport into daily commutation. Although physical activity is considered essential for the prevention and treatment of most chronic diseases, exercise is rarely prescribed for CKD (25). Progression of CKD leads to decreased number of steps walked (26) and decreased physical activity leads to increased incidence of CKD, albuminuria (27). Therefore, we also aim to obtain scientific evidence that daily physical activities for health have a positive impact on CKD by city planning.

Another issue in rural city planning is the medical system. There is a link between the detrimental progression of CKD and access to specialized medical care (28). In such rural areas, due to rural depopulation and lack of specialized healthcare providers, medical supply concentrations are so low that the government has recognized these areas as major projects to designate hospitals for integration (29). In our experience in a rural district, a population of one hundred thousand and had already undergone hospital integration, screening for detecting advanced CKD patients with high-risk was beneficial in the delegation of appropriate referrals from generalists to nephrologists and/or cardiologists (11). In this investigation, we developed an algorithm to detect CKD in an electronic medical record, among retrospectively obtained data from April 2018 to December 2019. From 12,335 men and 9,756 women, we were able to collect data of 61,588 creatinine measurements and 40,973 dipstick proteinuria tests over the integration. The sequential data from individual patients sometimes contained transient elevations of serum creatinine. To exclude patients with acute kidney injury (AKI) in this study, the latest data were also assessed to determine whether renal injury had recovered from eGFR less than 30 mL/min/1.73 m^2^ to more than 30 mL/min/1.73 m^2^. If a subject did not have AKI, he or she was regarded as possibly having CKD (11). Based on the procedure, we found that 108 had AKI, 133 were on chronic dialysis, 133 died before the end of the investigation, and 76 were transferred to other facilities or were started on conservative terminal care without the option of having chronic dialysis. This screening finally identified29 (0.13%) patients with nondialysis CKD G5 and 62 (0.28%) patients with CKD G4 and with massive proteinuria, who have high possibility of future introduction of renal replacement therapy (11). With a number of renal failure patients of this size, the induction of renal replacement therapy or conservative kidney management may be possible with a single nephrologist under conditions of adequate coordination of medical care. In other words, this requires a general practitioner’s practice contribution in the early stage of CKD and the nephrologist can focus on advanced CKD patients. In addition, we foresee distance and convenience as barriers for management of advanced CKD patients in integrated hospitals for the visit for each patient. As long as the current access to core hospitals in rural cities is mainly by private vehicle, many may continue to experience the frustrations of traffic congestion and lack of parking. Therefore, the availability of public transportation and the location of hospital integration should be fully considered.

Recently, special consideration must be given to the use of public transportation since there is the issue of epidemic infectious diseases, including COVID-19. Public transport accessibility—one strength of a compact city—has negative links to some health and well-being outcomes during the COVID-19 pandemic, possibly due to increased risk of infection and related stress (30). To mitigate this issue, public transport departures need to be frequent enough to guarantee safe social distancing during pandemic crises. In addition, alternative transport options need to be available in compact, transit-oriented neighborhoods. If public transportation infrastructure is difficult to put in place for some reason, one might consider using a combination of telehealth techniques, because telehealth has become a valuable tool to leverage specialized medical care by nephrologist with equally high satisfaction compared to face-to face manner during COVID-19 pandemic (31).

## Discussion

>Evaluating the monetary index has the potential to use market mechanisms to put pressure on companies and societies to change the policy of industry and city planning (32). Therefore, we describe the possible effects on the economy by city planning at the end (**Figure 1**). Even though social demonstration experiments on physical activity to attain better health are being conducted, they are only in limited areas and on a limited scale (33). While there have been many examinations of the economic benefits of integrating hospitals, few reports show the effect on prognosis for specific diseases. From this perspective, we have focused on the possible links between city planning and intermediate factors of developing CKD to improve renal health in Japan. Interventional investigations on physical activity and medical expenditure suggest that undergoing sufficient levels of physical activity is associated with decreased healthcare costs (33, 34). In the Japanese middle-aged population, a one-step-increase in the annual average daily step count reduced outpatient healthcare costs by 16.26 JPY in the short run and assumed long-run effects of daily steps were estimated at 28.24 JPY (33). Regarding preserving renal function and CKD deterioration, decline speed in eGFR is considered as a surrogate marker for ESKD. In the male general population, the annual costs for low eGFR subjects with a rapid decrease in eGFR (741 thousand JPY) were more than twice those of non-low eGFR subjects with a rapid decrease in eGFR (349 thousand JPY) and also larger compared to low eGFR subjects with a stable eGFR (336 thousand JPY) (35). We hope to maintain renal health by educating a wide range of people on the potential increase in financial burden that physical inactivity and regional disparities of renal healthcare that arises from inappropriate city planning.

Many countries are concerned by the costs associated with the mounting burden of lifestyle-related chronic disease (36). They have put in place plans and public policy initiatives that encourage increased levels of physical activity. In June 2020 in Japan, the Law Concerning Special Measures for Urban Revitalization was amended to establish a location optimization planning system to promote the formation of compact cities (37). Under this system, local governments can establish two types of guidance zones, residential and urban. Residential guidance zones concentrate residential property and urban function guidance zones concentrate stores and welfare facilities, thereby creating efficient cities. We should focus on the impact of these community initiatives on CKD, the well-being and convenience of the residents, and the economic benefits.

## Data Availability Statement

The raw data supporting the conclusions of this article will be made available by the authors, without undue reservation.

## Ethics Statement

The studies involving human participants were reviewed and approved by Hitachi General Hospital. Written informed consent for participation was not required for this study in accordance with the national legislation and the institutional requirements.

## Author Contributions

The corresponding author (KN) designed this investigation and data review with supervision by AU. DC reviewed and edited the manuscript. All authors contributed to the article and approved the submitted version.

## Funding

This article was supported, in part, by JSPS Grant Nos. 18KK0431 and 19K17729 and by the Japanese Association of Dialysis Physicians Grant No. 2020-3.

## Conflict of Interest

The authors declare that the research was conducted in the absence of any commercial or financial relationships that could be construed as a potential conflict of interest.

## Publisher’s Note

All claims expressed in this article are solely those of the authors and do not necessarily represent those of their affiliated organizations, or those of the publisher, the editors and the reviewers. Any product that may be evaluated in this article, or claim that may be made by its manufacturer, is not guaranteed or endorsed by the publisher.

## References

[1] GBD Chronic Kidney Disease Collaboration. Global, Regional, and National Burden of Chronic Kidney Disease, 1990-2017: A Systematic Analysis for the Global Burden of Disease Study 2017. Lancet (2020) 395(10225):709–33. doi: 10.1016/S0140-6736(19)32977-0 PMC7049905 32061315

[2] Couser WG Remuzzi G Mendis S Tonelli M. The Contribution of Chronic Kidney Disease to the Global Burden of Major Noncommunicable Diseases. Kidney Int (2011) 80(12):1258–70. doi: 10.1038/ki.2011.368 21993585

[3] Nagai K Asahi K Iseki K Yamagata K. Estimating the Prevalence of Definitive Chronic Kidney Disease in the Japanese General Population. Clin Exp Nephrol (2021) 25(8):885–92. doi: 10.1007/s10157-021-02049-0 33839966

[4] Perlman RL Finkelstein FO Liu L Roys E Kiser M Eisele G. Quality of Life in Chronic Kidney Disease (CKD): A Cross-Sectional Analysis in the Renal Research Institute-CKD Study. Am J Kidney Dis (2005) 45(4):658–66. doi: 10.1053/j.ajkd.2004.12.021 15806468

[5] Cockwell P Fisher LA. The Global Burden of Chronic Kidney Disease. Lancet (2020) 395(10225):662–4. doi: 10.1016/S0140-6736(19)32977-0 32061314

[6] Johansen KL Painter P. Exercise in Individuals With CKD. Am J Kidney Dis (2012) 59(1):126–34. doi: 10.1053/j.ajkd.2011.10.008 PMC3242908 22113127

[7] Blair SN. Physical Inactivity: The Biggest Public Health Problem of the 21st Century. Br J Sports Med (2009) 43(1):1–2. 19136507

[8] Ding D Lawson KD Kolbe-Alexander TL Finkelstein EA Katzmarzyk PT van Mechelen W. The Economic Burden of Physical Inactivity: A Global Analysis of Major non-Communicable Diseases. Lancet (2016) 388(10051):1311–24. doi: 10.1016/S0140-6736(16)30383-X 27475266

[9] Ikeda N Inoue M Iso H Ikeda S Satoh T Noda M. Adult Mortality Attributable to Preventable Risk Factors for non-Communicable Diseases and Injuries in Japan: A Comparative Risk Assessment. PloS Med (2012) 9(1):e1001160. doi: 10.1371/journal.pmed.1001160 22291576 PMC3265534

[10] Hoshino J Muenz D Zee J Sukul N Speyer E Guedes M. Associations of Hemoglobin Levels With Health-Related Quality of Life, Physical Activity, and Clinical Outcomes in Persons With Stage 3-5 Nondialysis CKD. J Ren Nutr (2020) 30(5):404–14. doi: 10.1053/j.jrn.2019.11.003 31980326

[11] Nagai K Hosoi T Nakajima K Arai T Nakamura Y. Screening for Chronic Kidney Disease Over Hospital Integration. J Gen Fam Med (2020) 21(6):294–5. doi: 10.1002/jgf2.375 PMC7689234 33304733

[12] Tsuchida-Nishiwaki M Uchida HA Takeuchi H Nishiwaki N Maeshima Y Saito C. Association of Blood Pressure and Renal Outcome in Patients With Chronic Kidney Disease; a *Post Hoc* Analysis of FROM-J Study. Sci Rep (2021) 11(1):14990. doi: 10.1038/s41598-021-94467-z 34294784 PMC8298520

[13] Yamagata K Makino H Iseki K Ito S Kimura K Kusano E. Effect of Behavior Modification on Outcome in Early- to Moderate-Stage Chronic Kidney Disease: A Cluster-Randomized Trial. PloS One (2016) 11(3):e0151422. doi: 10.1371/journal.pone.0151422 26999730 PMC4801411

[14] Giles-Corti B Vernez-Moudon A Reis R Turrell G Dannenberg AL Badland H. City Planning and Population Health: A Global Challenge. Lancet (2016) 388(10062):2912–24. doi: 10.1016/S0140-6736(16)30066-6 27671668

[15] Mouratidis K. Urban Planning and Quality of Life: A Review of Pathways Linking the Built Environment to Subjective Well-Being. Cities (2021) 103229:1–12. doi: 10.1016/j.cities.2021.103229

[16] Hitachi General Hospital. . Available at: https://www.hitachi.co.jp/hospital/hitachi/bumon/lifestyle/index.html (Accessed Apr 1, 2022).

[17] Ballin M Nordström P Niklasson J Alamäki A Condell J Tedesco S. Daily Step Count and Incident Diabetes in Community-Dwelling 70-Year-Olds: A Prospective Cohort Study. BMC Public Health (2020) 20(1):1830. doi: 10.1186/s12889-020-09929-2 33256704 PMC7706282

[18] Kraus WE Yates T Tuomilehto J Sun JL Thomas L McMurray JJV. Relationship Between Baseline Physical Activity Assessed by Pedometer Count and New-Onset Diabetes in the NAVIGATOR Trial. BMJ Open Diabetes Res Care (2018) 6(1):e000523. doi: 10.1136/bmjdrc-2018-000523 PMC6067333 30073088

[19] Ponsonby AL Sun C Ukoumunne OC Pezic A Venn A Shaw JE. Objectively Measured Physical Activity and the Subsequent Risk of Incident Dysglycemia: The Australian Diabetes, Obesity and Lifestyle Study (AusDiab). Diabetes Care (2011) 34(7):1497–502. doi: 10.2337/dc10-2386 PMC3120195 21562319

[20] Cochrane SK Chen SH Fitzgerald JD Dodson JA Fielding RA King AC. Association of Accelerometry-Measured Physical Activity and Cardiovascular Events in Mobility-Limited Older Adults: The LIFE (Lifestyle Interventions and Independence for Elders) Study. J Am Heart Assoc (2017) 6(12):e007215. doi: 10.1161/JAHA.117.007215 29197830 PMC5779035

[21] Huffman KM Sun JL Thomas L Bales CW Califf RM Yates T. Impact of Baseline Physical Activity and Diet Behavior on Metabolic Syndrome in a Pharmaceutical Trial: Results From NAVIGATOR. Metabolism (2014) 63(4):554–61. doi: 10.1016/j.metabol.2014.01.002 PMC4103164 24559843

[22] Yates T Haffner SM Schulte PJ Thomas L Huffman KM Bales CW. Association Between Change in Daily Ambulatory Activity and Cardiovascular Events in People With Impaired Glucose Tolerance (NAVIGATOR Trial): A Cohort Analysis. Lancet (2014) 383(9922):1059–66. doi: 10.1016/S0140-6736(13)62061-9 24361242

[23] Frank LD Adhikari B White KR Dummer T Sandhu J Demlow E. Chronic Disease and Where You Live: Built and Natural Environment Relationships With Physical Activity, Obesity, and Diabetes. Environ Int (2022) 158:106959. doi: 10.1016/j.envint.2021.106959 34768046

[24] Hajna S Ross NA Brazeau AS Bélisle P Joseph L Dasgupta K. Associations Between Neighbourhood Walkability and Daily Steps in Adults: A Systematic Review and Meta-Analysis. BMC Public Health (2015) 15:768. doi: 10.1186/s12889-015-2082-x 26260474 PMC4532296

[25] Barcellos FC Santos IS Umpierre D Bohlke M Hallal PC. Effects of Exercise in the Whole Spectrum of Chronic Kidney Disease: A Systematic Review. Clin Kidney J (2015) 8(6):753–65. doi: 10.1093/ckj/sfv099 PMC4655802 26613036

[26] Zhang F Ren Y Wang H Bai Y Huang L. Daily Step Counts in Patients With Chronic Kidney Disease: A Systematic Review and Meta-Analysis of Observational Studies. Front Med (Lausanne) (2022) 9:842423. doi: 10.3389/fmed.2022.842423 35252275 PMC8891233

[27] Kelly JT Su G Zhang L Qin X Marshall S González-Ortiz A. Modifiable Lifestyle Factors for Primary Prevention of CKD: A Systematic Review and Meta-Analysis. J Am Soc Nephrol (2021) 32(1):239–53. doi: 10.1681/ASN.2020030384 PMC7894668 32868398

[28] Bello AK Hemmelgarn B Lin M Manns B Klarenbach S Thompson S. Impact of Remote Location on Quality Care Delivery and Relationships to Adverse Health Outcomes in Patients With Diabetes and Chronic Kidney Disease. Nephrol Dial Transplant (2012) 27(10):3849–55. doi: 10.1093/ndt/gfs267 22759385

[29] Ministry of Health, Labour and Welfare. . Available at: https://www.mhlw.go.jp/content/10800000/000551037.pdf (Accessed Apr 1, 2022).

[30] Mouratidis K. COVID-19 and the Compact City: Implications for Well-Being and Sustainable Urban Planning. Sci Total Environ (2022) 811:152332. doi: 10.1016/j.scitotenv.2021.152332 34914991 PMC8666382

[31] Androga LA Amundson RH Hickson LJ Thorsteinsdottir B Garovic VD Manohar S. Telehealth Versus Face-to-Face Visits: A Comprehensive Outpatient Perspective-Based Cohort Study of Patients With Kidney Disease. PloS One (2022) 17(3):e0265073. doi: 10.1371/journal.pone.0265073 35275958 PMC8916620

[32] Inaba A Itsubo N. Preface. Int J Life Cycle Assess (2018) 23:2271–5. doi: 10.1007/s11367-018-1545-6

[33] Okamoto S Kamimura K Shiraishi K Sumita K Komamura K Tsukao A. Daily Steps and Healthcare Costs in Japanese Communities. Sci Rep (2021) 11(1):15095. doi: 10.1038/s41598-021-94553-2 34301997 PMC8302729

[34] Yoshizawa Y Kim J Kuno S. Effects of a Lifestyle-Based Physical Activity Intervention on Medical Expenditure in Japanese Adults: A Community-Based Retrospective Study. BioMed Res Int (2016) 2016:7530105. doi: 10.1155/2016/7530105 27493963 PMC4963587

[35] Nagai K Iseki C Iseki K Kondo M Asahi K Saito C. Higher Medical Costs for CKD Patients With a Rapid Decline in eGFR: A Cohort Study From the Japanese General Population. PloS One (2019) 14(5):e0216432. doi: 10.1371/journal.pone.0216432 31100069 PMC6524806

[36] Murray CJ Vos T Lozano R Naghavi M Flaxman AD Michaud C. Disability-Adjusted Life Years (DALYs) for 291 Diseases and Injuries in 21 Regions, 1990-2010: A Systematic Analysis for the Global Burden of Disease Study 2010. Lancet (2012) 380(9859):2197–223. doi: 10.1016/S0140-6736(12)61689-4 23245608

[37] Ministry of Land, Infrastructure, Transport and Tourism. Available at: https://www.mlit.go.jp/en/toshi/index.html (Accessed Apr 1, 2022).

